# Broad Echo State Network with Reservoir Pruning for Nonstationary Time Series Prediction

**DOI:** 10.1155/2022/3672905

**Published:** 2022-02-27

**Authors:** Wenjie Liu, Yuting Bai, Xuebo Jin, Xiaoyi Wang, Tingli Su, Jianlei Kong

**Affiliations:** ^1^School of Artificial Intelligence, Beijing Technology and Business University, Beijing 100048, China; ^2^Beijing Laboratory for Intelligent Environmental Protection, Beijing Technology and Business University, Beijing 100048, China; ^3^State Environmental Protection Key Laboratory of Food Chain Pollution Control, Beijing Technology and Business University, Beijing 100048, China

## Abstract

The nonstationary time series is generated in various natural and man-made systems, of which the prediction is vital for advanced control and management. The neural networks have been explored in the time series prediction, but the problem remains in modeling the data's nonstationary and nonlinear features. Referring to the time series feature and network property, a novel network is designed with dynamic optimization of the model structure. Firstly, the echo state network (ESN) is introduced into the broad learning system (BLS). The broad echo state network (BESN) can increase the training efficiency with the incremental learning algorithm by removing the error backpropagation. Secondly, an optimization algorithm is proposed to reduce the redundant information in the training process of BESN units. The number of neurons in BESN with a fixed step size is pruned according to the contribution degree. Finally, the improved network is applied in the different datasets. The tests in the time series of natural and man-made systems prove that the proposed network performs better on the nonstationary time series prediction than the typical methods, including the ESN, BLS, and recurrent neural network.

## 1. Introduction

Time series data is observed and measured over time in human society and the natural environment. The analysis and prediction of the time series data have drawn attention because it is vital for managing and controlling various man-made and natural systems. For example, the prediction of sales data is applied to optimize inventory and reduce social costs [1]. The stock data prediction can foresee the capital flows trend [2]. The precipitation [3], water bloom [4], and typhoon intensity are also predicted for natural environment protection and disaster prevention [5]. The trend forecast of air pollutants provides strong support for the decision-making of relevant departments in the future [6–8]. The nonstationary and nonlinear trend has been the obvious feature of time series data in various application contexts. It is impossible to extract and represent data trends intuitively because the change rule of the time series is stochastic and complex. It has been a research issue how to extract the data features and predict the future trend of the time series.

For the mainstream of time series prediction, there are statistical methods [9–11] and machine learning methods [12–16]. Machine learning includes external neural networks, deep learning networks, and broad learning system (BLS). Statistical prediction methods mainly include the autoregressive (AR) model, moving average (MA) model, autoregressive moving average (AR-MA) model, and differential autoregressive moving average (ARIMA) model integration, etc. They transform nonstationary time series into stationary time series utilizing variance or integration. They face application difficulty when dealing with nonstationary and nonlinear time series in existing systems because the value of the loss function is hard to reduce with the solution of stationary data transformation. Machine learning has been widely used with a novel modeling solution, which focuses on the data feature with a black-box model. It uses nonlinear algorithms to reduce the loss function with a network structure [17]. It can also utilize the computing ability of the hardware to improve model accuracy, minimize loss function value, and improve network fitting ability. The typical machine learning methods include support vector machines, decision trees, recursive neural networks, convolutional neural networks, BLS, etc. For the representative and hotspot methods, deep learning networks require large-scale computing resources, although they improve prediction performance. BLS reduces the consumption of computer resources under the same prediction accuracy [12]. Meanwhile, the echo state network (ESN) [18] has been proven effective in time series modeling with a simple training algorithm. Therefore, it is explored to utilize the advantages of the different machine learning methods, including their structure and learning algorithm.

In the literature research and previous studies, it is found that the performance improvement relies on the network structure expansion and computing resources occupation. A solution should be explored with an appropriate network scale and a fast learning algorithm. The ESN does not need to update backpropagation parameters because only the internal weighting matrix should be updated. In contrast, the input and reservoir pool weight matrixes need not be updated. The learning mechanism of ESN is highly efficient. The BLS builds a horizontal network instead of the vertical network of deep learning. The parameter passing can be obviously improved without the progression of multiple layers. Therefore, referring to the advantages of ESN and BLS, a novel network of broad echo state networks (BESN) is designed in this paper to predict nonstationary time series. The BESN has been mentioned in [19], in which the raw data are imported into multiple parallel running reservoir pools with the unsupervised learning algorithm of the restricted Boltzmann machine. It mainly arranges the echo reservoirs parallelly, and there are no concepts of mapping and enhancement layers of BLS. In this paper, BESN is a combination of ESN and BLS. The enhancement layer of the BLS is remolded with ESNs in this paper. Meanwhile, the redundant nodes may exist in ESN, and a pruning optimization algorithm is introduced. Then, the broad pruning echo state network (BPESN) is finally established in this paper.

This paper is organized as follows. The second section introduces related work of time series analysis and prediction method. The third section introduces the main study of BPESN proposed in this paper. The fourth section presents the experimental environment and experiments. The fifth section introduces the experimental results and analysis. The sixth section is the summary of this paper.

## 2. Related Works

### 2.1. Analysis of Nonstationary Time Series Data

A nonstationary time series can be described with the statistical variates, in which the mean and covariance of the data changes dynamically over time [20]. The nonstationary trend has been the typical characteristic of the time series data, especially for existing systems. The inherent complexity of actual data can be represented with the nonstationary indexes [21, 22]. The nonstationary degree analysis is the basis for the time series modeling and prediction.

There are many methods to test the stationarity of time series data, among which the most commonly used are the correlation test [23] and the Augmented Dickey-Fuller (ADF) test [24]. The correlation test determines whether there is trailing and truncation through the correlation function of the time series as the basis for judging whether the data is stable. The ADF test is a more scientific judgment method based on whether the mean and variance of time series change over time. Meanwhile, probability values (*p*), test statistics (*TS*), 1% critical value (*CV*_1_), 5% critical value (*CV*_5_), and 10% critical value (*CV*_10_) will be generated in a standard test. The stability of the time series can be judged by judging *p* and the relationship between the test statistics and the critical value.

The null hypothesis of ADF is that the detection sequence has a unit root and is a nonstationary time series. When *p* < 0.05, the null hypothesis is rejected, and the time series is stationary [25]. According to the Akaike information criterion (AIC), the test time series is nonstationary when *p* ≥ 0.05, *TS* > *CV*_1_, *TS* > *CV*_5_, *TS* > *CV*_10_, and the null hypothesis is not denied.

### 2.2. Time Series Prediction Method

Early time series modeling mainly used statistical methods. Based on the randomness theory of time series, the AR model [26] and MA model [27] are proposed. The AR model uses the correlation between the previous data and the later data to establish a regression equation containing the previous data and the later data. The MA model solves the problem of summing the white noise of the AR model and organically combines the MA model and the AR model to form the ARMA model [28]. The ARMA model energizes the correlation between the current data and the previous data and at the same time can solve the problem of randomly changing items. ARIMA is commonly used in nonstationary time series [10]. ARIMA transforms nonstationary time series into stationary time series through the difference between adjacent time points. Machine learning closely follows the era of big data. It makes full use of computer configuration and gives birth to many typical neural network models, such as the Back Propagation (BP) model [29], Long Short-Term Memory (LSTM) model [30], Gated Recurrent Unit (GRU) model [31], BLS model, etc. The BP model is a multilayer neural network that propagates the error back and updates the weight matrix continuously. LSTM and GRU are specifically born in RNN to solve the problem of short-term memory. They can also solve the problem of gradient disappearance and gradient explosion in RNN to a certain extent. Both LSTM and GRU have internal gates, which are used to regulate information flow. The difference between them lies in the number of gates. Compared with LSTM, GRU consumes less computing resources when achieving the same accuracy. BLS is different from deep learning, and the network structure is not deepened vertically but expanded horizontally and consumes more computer resources [12].

Statistical methods can accurately realize the modeling of stationary time series, but the effect will be poor when the data is nonstationary and nonlinear with violent fluctuations. Machine learning applies to the nonstationary time series with the network structure and the computing resources. When time series data is large, the machine learning model itself will be significantly affected. ESN and BLS can effectively deal with the problem that large datasets cause large computer resource consumption.

### 2.3. Echo State Network

**Figure 1:**
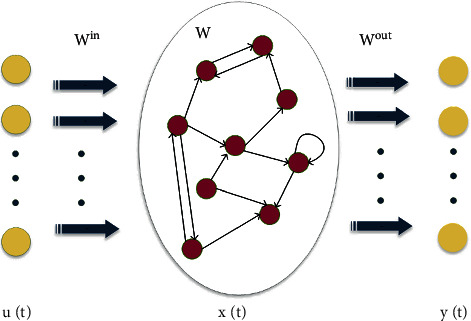
The structure of ESN (Figure 1 is reproduced from Liu et al. [33]).

ESN is a recurrent neural network that consists of three parts: input layer, reservoir pool, and output layer. It can map the input data to high dimensions in the reservoir pool with an input weighting matrix [32]. ESN simplifies the training task of the network, in which only the weights of the output matrix should be trained without the traditional backpropagation. The topology structure of ESN is shown in Figure 1[33].

In Figure 1, the input is defined as **u**(*t*)=[*u*_1_(*t*), *u*_2_(*t*),…, *u*_*K*_(*t*)], the reservoir pool's state **x**(*t*)=[*x*_1_(*t*), *x*_2_(*t*),…, *x*_*N*_(*t*)], and the network output is **y**(*t*)=[*y*_1_(*t*), *y*_2_(*t*),…, *y*_*L*_(*t*)]. *K*, *N*, *L* represent the number of input samples, the number of neurons in the reservoir pool, and the output dimension. To ensure the echo characteristics of the reservoir pool, the spectral radius should be set from 0 to 1. The status update and network output of the reservoir pool are as follows:

(1) $$\begin{array}{c} x\left(t+1\right)=\left(1-\alpha \right)x\left(t\right)+\alpha \cdot f\left(W^{in}u\left(t\right)+Wx\left(t\right)\right),\\ y\left(t+1\right)=g\left(W^{out}x\left(t+1\right)\right), \end{array}$$

where **x**(*t*+1) represents the state vector of the reservoir pool at *t*+1, **y**(*t*+1) means the output of the model at *t*+1, *α* represents the leakage coefficient of the reservoir pool, ranging from 0 to 1, **W**^*out*^ ∈ *ℝ*^*N*×*N*^ represents the output weight matrix, *f*(•), *g*(•), respectively, represent the activation function of the reservoir pool and the output layer.

**W** ^*in*^, **W** in ESN are randomly generated and remain unchanged during training and testing, so the only thing that needs to be adjusted during the learning process is **W**^*out*^. There are many solving methods for **W**^*out*^, including ridge regression [34], recursive least square method [35], pseudo-inverse method [36], and singular value decomposition method. In this paper, ridge regression is selected to solve **W**^*out*^. The ridge regression is a biased estimation method, essentially a modified least-squares estimation method. Ridge regression is abandoned to obtain higher computational accuracy, unlike the unbiased method. It will be more suitable for the discomfort problem with a pathological matrix or pathological solution. The reservoir pool in ESN has a pathological resolution due to sparse connection, so the ridge regression is selected to solve **W**^*out*^. The calculation of **W**^*out*^ is as follows:

(2) $$\begin{array}{c} W^{out}=YX^{T}{\left(XX^{T}+\lambda Ι\right)}^{-1}, \end{array}$$

where **X** ∈ *ℝ*^*N*×*K*^ represents the state matrix of the reservoir pool and **Y** ∈ *ℝ*^*L*×*K*^ represents the actual output of the model, *λ* is a regularization coefficient, and **I** is the identity matrix. It is advisable to set the first element of **I** into zero to exclude the bias connection from the regularization [37].

Since ESN was proposed in 2001, it has been widely used in time prediction. However, the disadvantage of ESN is that many parameter selections are realized by trial and error method, and it cannot effectively learn an intelligent choice of models based on different data. Therefore, many scholars optimize the design of its network structure. For example, Sheng C et al. proposed an improved version adding noise to the ESN network [38], and Jun xu Liu et al. proposed quantum-based ESN [39]. Among many parameters of the ESN model, the selection of reservoir pool size is significant. In this paper, pruning neurons in a single reservoir pool is performed by a pruning optimization algorithm. After several cuts, the reservoir pool will reach the desired state.

### 2.4. Broad Learning System

BLS is a forward neural network based on a random vector function connection network [12] and an efficient machine learning method. The network structure of BLS includes the mapping layer, enhancement layer, and output layer. Compared with the random vector function connection network, the mapping layer replaces the output layer. In BLS, the original data are imported into the linear mapping and become a node mapping layer. It realizes the feature extraction of the original data. The mapping layer increases the model's ability to adapt to the nonlinear data. Finally, the combined matrix of the mapping layer's output and the enhancement layer's output is used as the input of BLS. The ridge regression algorithm obtains the output weight matrix. The output of the mapping layer and the output of the enhancement layer are as follows:

(3) $$\begin{array}{c} Z_{j}=\phi \left(XW_{ej}+\beta_{ej}\right),j=1,2,\ldots ,n,\\ H_{i}=\delta \left(ZW_{hi}+\beta_{hi}\right),i=1,2,\ldots ,m, \end{array}$$

where **W**_*ej*_, **W**_*hi*_ represent the weight matrix of the mapping layer and enhancement layer, *β*_*ej*_, *β*_*hi*_, respectively, represent the bias of mapping layer and enhancement layer, **W**_*ej*_, **W**_*hi*_, *β*_*ej*_, *β*_*hi*_ which are randomly generated, *n*, *m*, respectively, represent the number of network input samples and the number of neurons of mapping layer.

We assume **A**^*m*^ that it is the combination of the mapping layer's output matrix and the enhancement layer's output matrix. We assume **Z**=[**Z**_1_, **Z**_2_,…, **Z**_*n*_]**H**=[**H**_1_, **H**_2_,…, **H**_*m*_] that the output weight matrix can adopt the pseudo-inverse method commonly used in the neural network to solve the output weight matrix. However, when the input sample has a high capacity and requires a high training speed of the network, the pseudo-inverse method generally cannot meet the requirements [40], so regularization can be used to solve the output weight matrix indirectly [13]. The network output and output weight matrix are shown in the following:

(4) $$\begin{array}{c} Y=\left[Z_{1},Z_{2},\ldots ,Z_{n}|H_{1},H_{2},\ldots ,H_{m}\right]W^{\text{out}}\\ =\left[Z|H\right]W^{\text{out}}\\ =A^{m}W^{\text{out}}\\ W^{\text{out}}={\left(\lambda I+A^{m}{\left(A^{m}\right)}^{T}\right)}^{-1}{\left(A^{m}\right)}^{T}\mathrm{Y,} \end{array}$$

where **Y** represents network output, **W**^*out*^ represents weight matrix of network output, and *λ* represents regularization constraint item coefficient.

## 3. Broad Echo State Network with Reservoir Pruning

### 3.1. Network Structure and Learning Method

As mentioned in Introduction and Related Works, ESN reduces the training complexity of the network with a reservoir structure. BLS can improve the data fitting ability by extending the network units. The network scale should not be too large in the ideal network solution for nonstationary time series prediction, and the training algorithm should be fast. Then the BLS are explored to be combined with ESN.

**Figure 2:**
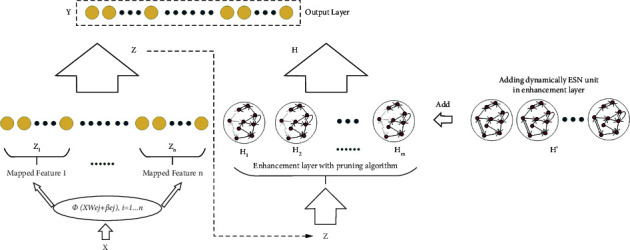
The structure of BPESN.

Meanwhile, the structure of ESN brings the redundancy nodes in the network, and the network should be optimized for a concise and effective structure. Concretely, neurons in the reservoir pool within each ESN are sparsely connected, resulting in different contributions to the entire network. The connection between the reservoir pool and the output layer is fully bonded, contrary to the sparse link of neurons in the reservoir pool [41]. Referring to the sparse connection in the reservoir pool, the redundant information may occur with the needless nodes and connected weights. The pruning algorithm is introduced into the ESN in the broad framework. The correlation between the reservoir pool of neurons is calculated, and the high correlation between sets of neurons connected weights is set to zero. The rest of the output weight is recalculated by the regression algorithm. The network structure proposed in this paper is shown in Figure 2.

As shown in Figure 2, the original data is extracted by linear mapping in the mapping layer to extract data features. The output of the mapping layer is used as the input of the enhancement layer. Each unit of the enhancement layer is ESN. The network output can be obtained through the combination matrix of the enhancement layer's output and the mapping layer's output. The effect is not up to the expected value. The number of ESNs is adjusted through the incremental algorithm, and the pruning optimization algorithm optimizes each ESN to optimize the network model. The output matrices of the mapping layer and the enhancement layer are **Z**=[**Z**_1_, **Z**_2_,…, **Z**_*n*_], **H**=[**H**_1_, **H**_2_,…, **H**_*m*_]. The output formula of each unit of the enhancement layer is as follows:

(5) $$\begin{array}{c} H_{i}\left(k\right)=\left(1-\alpha \right)H_{i}\left(k-1\right)+\alpha \delta \left(W_{hi}Z\left(k\right)+W_{i}H_{i}\left(k-1\right)\right). \end{array}$$

The enhancement layer begins to have the incremental learning ability by replacing the unit with ESN. The entire network can adapt to time series data and extract the data feature by dynamically adding an ESN unit until the network error reaches a default value. The update calculation is as follows:

(6) $$\begin{array}{c} A^{m+1}=\left[A^{m}|H_{m+1}\right],\\ {\left(A^{m+1}\right)}^{*}=\left[\begin{array}{c} {\left(A^{m}\right)}^{*}-DB^{T}\\ B^{T} \end{array}\right],\\ W^{\prime }=\left[\begin{array}{c} W-DB^{T}Y\\ B^{T}Y \end{array}\right]. \end{array}$$

where **A**^*m*^ is the state matrix, (**A**^*m*^)^*∗*^ is the pseudo inverse matrix of **A**^*m*^, **W**′ is the output weight matrix after adding the ESN unit, **Y** is the output of the network, and **B**, **C**, and **D** are defined as follows:

(7) $$\begin{array}{c} D={\left(A^{m}\right)}^{*}H_{m+1},\\ B^{T}=\left\{\begin{array}{c} {\left(C\right)}^{*},C\neq 0,\\ {\left(1+D^{T}D\right)}^{-1}B^{T}{\left(A^{m}\right)}^{*},C=0, \end{array}\right.\\ C=H_{m+1}-A^{m}D. \end{array}$$

The thresholds should be set in adding ESN units adaptively, and the thresholds can be determined with Root Mean Squared Error (RMSE) and Mean Absolute Deviation (MAE). It need not recalculate the pseudo inverse (**A**^*m*^)^*∗*^ of the state matrix in each time of adding an ESN. It only needs to update the ESN based on the previous network parameters, significantly shortening the running time.

The correlation between neurons in the reservoir pool of each ESN unit is calculated, and the correlation matrix is obtained, as follows:

(8) $$\begin{array}{c} r_{nm}=\frac{\sum_{i=1}^{T}\left(y_{ni}-{\bar{y}}_{n}\right)\left(y_{mi}-{\bar{y}}_{m}\right)}{\sqrt{{\sum_{i=1}^{T}\left(y_{ni}-{\bar{y}}_{n}\right)}^{2}{\sum_{i=1}^{T}\left(y_{mi}-{\bar{y}}_{m}\right)}^{2}}},\\ {\bar{y}}_{n}=\frac{1}{N}\overset{N}{\underset{i=1}{\sum }}y_{ni}, \end{array}$$

where *r*_*nm*_ represents the correlation coefficient between the *n*-th neuron and the *m*-th neuron in the reservoir pool, ${\bar{y}}_{n}$ represents the mean value of the state vector of the layer where the neuron in the *N* reservoir pool resides, *T* represents the number of state vectors, and *N* represents the number of neurons in the reservoir pool.

According to the correlation coefficient, the correlation matrix **r** of a single ESN unit can be determined as follows:

(9) $$\begin{array}{c} r=\left[\begin{array}{cccc} r_{11} & r_{12} & \cdots & r_{1N}\\ r_{21} & r_{22} & \cdots & r_{2N}\\ \vdots & \vdots & \ddots & \vdots \\ r_{N1} & r_{N2} & \cdots & r_{NN} \end{array}\right]. \end{array}$$

Based on the correlation matrix, the subscripts of *k* sets of the largest elements in **r** are denoted as **S**=[(*s*_*n*1_, *s*_*m*1_)_1_, (*s*_*n*2_, *s*_*m*2_)_2_,…, (*s*_*nk*_, *s*_*mk*_)_*k*_]. The neurons with the same serial number with the subscript in **S** are selected, and their weight matrix is set to zero. Then, the ESN is trained again, and the weights of the output matrix are calculated to obtain the network error.

### 3.2. Pruning Algorithm to Optimize the Network

**Figure 3:**
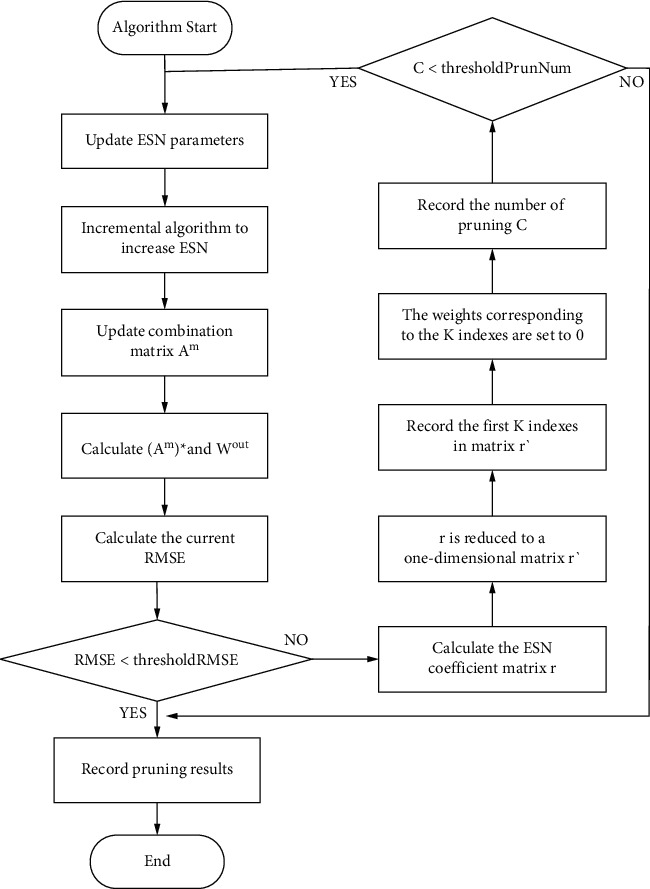
Pruning algorithm flowchart.

In this paper, each ESN in the enhancement layer is optimized to find the most appropriate size of the reservoir pool. The internal neuron connection of the ESN reservoir pool is sparse, and each neuron contributes to the network differently. The pruning optimization algorithm is a practical improvement to reduce the calculation cost, save time, and improve the accuracy to a certain extent. Algorithm optimization aims to calculate the correlation between the neurons in each reservoir pool, obtain the correlation matrix, and set the weight corresponding to the *k* neuron with the highest correlation to zero. From the experimental results, the pruning optimization algorithm can reduce the error of the network model. The algorithm flow is shown in Figure 3, and the overall algorithm is shown in Algorithm 1.

## 4. Experiments and Results

### 4.1. Experimental Settings and Datasets

#### 4.1.1. Experimental Environment and Settings

The experiments are designed to verify the nonstationary time series prediction neural network. Two different types of time series data are selected as the subject to be predicted. One dataset is the air quality monitoring data of Fangshan District in Beijing, and the other is the power load data of the United States. The two datasets represent the different systems, of which the air quality data is from the natural environment, and the power load data is from the man-made system. The data can be regarded as the typical time series in the common systems.

The experiments are conducted on a small computing platform. The platform is based on a 64-bit Windows system. Its RAM is 16 GB, and the processor is AMD R7 4800H (2.9 GHz). The deep learning framework is based on Tensorflow2.0. and Keras2.4.3. The code is applied in the programming language of Python 3.7.

In this paper, some typical time series prediction models are selected as contrast methods. The proposed method is BPESN, which integrates the ESN with BLS and the pruning optimization algorithm. As the basis of the proposed BPESN, the typical non-feedback neural network ESN and BLS are set as the contrast. Besides, the integrated structure of ESN and BLS is also set as the contrast, which is called BESN. Moreover, the recurrent neural network has been the representative method in the time series prediction. LSTM is selected on behalf of the feedback recurrent neural network. As an improvement of LSTM, GRU is widely applied in prediction, which is also set as the comparison model. Meanwhile, the k-fold cross-validation is a standard validation method for model evaluation and selection in the field of machine learning [42]. It aims at avoiding the occurrence of chance phenomena due to unreasonable data division. Then, this paper uses k-fold cross-validation for the evaluation of BPESN models to better evaluate the performance of network models.

Some quantitative indexes are selected to evaluate the prediction performance. The regression evaluation indexes of the chosen experimental model in this paper include Mean Absolute Deviation (MAE), Root Mean Squared Error (RMSE), Symmetric Mean Absolute Percentage Error (SMAPE), and determinant coefficients *R*^2^. MAE, RMSE, and SMAPE reflect the predicted and actual values deviation. The smaller the three values, the better the model performance. *R*^2^ reflects the reasonable degree of the final prediction model. The closer to 1 for *R*^2^, the better the fitting degree of the prediction model is. The formula of each evaluation index is as follows:

(10) $$\begin{array}{c} \text{MAE}=\frac{1}{n}\overset{n}{\underset{k=1}{\sum }}\left|y\left(k\right)-\hat{y}\left(k\right)\right|,\\ \text{RMSE}=\sqrt{\frac{\sum_{k=1}^{n}{\left(y\left(k\right)-\hat{y}\left(k\right)\right)}^{2}}{n}},\\ \text{SMAPE}=\frac{1}{n}\overset{n}{\underset{k=1}{\sum }}\frac{\left|y\left(k\right)-\hat{y}\left(k\right)\right|}{y\left(k\right)+\hat{y}\left(k\right)},\\ R^{2}=1-\frac{\sum_{i=1}^{n}{\left(y\left(k\right)-\hat{y}\left(k\right)\right)}^{2}}{\sum_{i=1}^{n}{\left(\hat{y}\left(k\right)-{\hat{y}}_{rv}\right)}^{2}}, \end{array}$$

where *n* is the number of samples, $\hat{y}\left(k\right)$ is the *k* − *th* predicted value, *y*(*k*) is the *k* − *th* actual value, and ${\hat{y}}_{rv}$ is the mean of the predicted values.

#### 4.1.2. Dataset

Two datasets are selected in the experiment, including the air quality monitoring dataset and the power load dataset.

**Figure 4:**
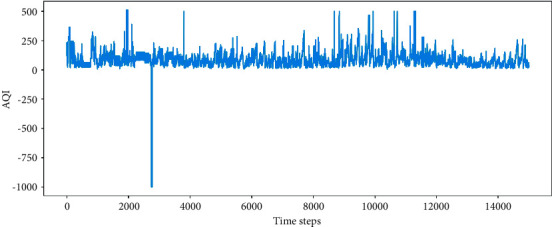
Air quality dataset of Fangshan District in Beijing.

The air quality monitoring dataset of the Fangshan District in Beijing includes AQI, CO, NO2, O3, PM10, PM2.5, and SQ2. For the evaluation of air quality, the air quality index (AQI) has been a comprehensive indicator calculated with concrete monitoring parameters. The data of AQI is analyzed in the experiment. The data are monitored and recorded hourly, which began on February 15, 2017, and ended on December 2, 2018. A total of 15,000 hours are covered. In the experiment, 12,000 test sets and 3,000 validation sets are included. The training sets account for 80% of the total samples, and the test sets account for 20%. The original trends of AQI data are shown in Figure 4.

**Figure 5:**
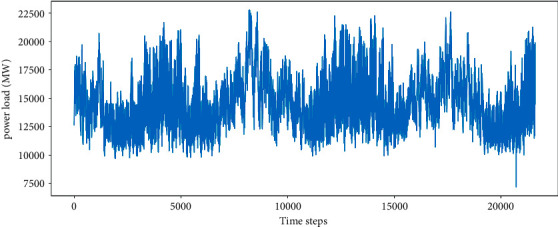
US power load dataset.

US electric power load data collection began on January 1, 2017, until January 1, 2020. The sampling interval is 1 hour. In this experiment, the continuous 900 days of data were selected as the total sample with 21600 sets. The training set accounts for 80% of the total sample number, and the test set accounts for 20%. The trend of the total samples is shown in Figure 5.

The nonstationary degree is tested firstly for the two datasets. The ADF tests are conducted to obtain the indicators. The test results are shown in Table 1.

It can be seen from Table 1 that the probability statistical value *p* of the air quality dataset and US power load dataset is greater than 0.05, and the TS is less than three critical values. According to the method in Section 2.1, the null hypothesis cannot be rejected in the ADF test; that is, the test sequence is nonstationary. It is concluded that both datasets in this experiment are nonstationary.

### 4.2. Prediction of Air Quality Monitoring Data

In the experiment, the concrete parameters of the networks are determined based on the data. The parameters of the networks in this test are shown in Table 2.

**Figure 6:**
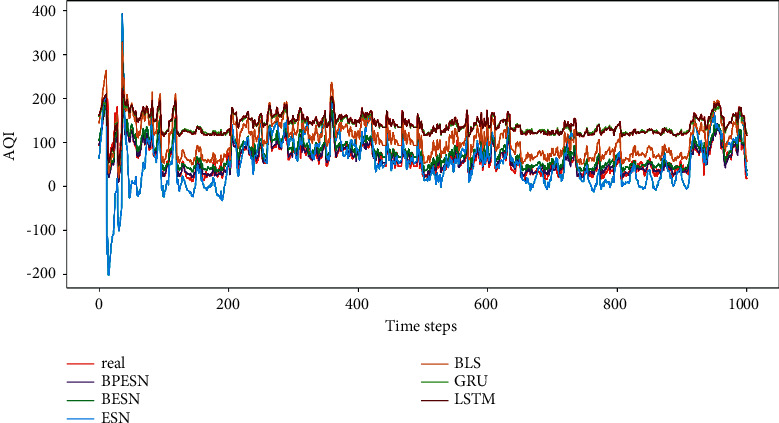
Prediction results of different network models in Fangshan District of Beijing.

The data of AQI is predicted with the proposed network, as well as the contrast methods. The results are shown in Figure 6, in which the results are denoted with lines in different colors. It can be found that the classical GRU and LSTM deviate from the actual value in the whole trend. Meanwhile, it can also be seen that ESN diverges significantly with the obvious fluctuation in nonstationary data. BESN will get better performance, which is combined with the advantages of BLS. BPESN fits the actual data curve most closely, based on BESN with the pruning optimization algorithm.

**Figure 7:**
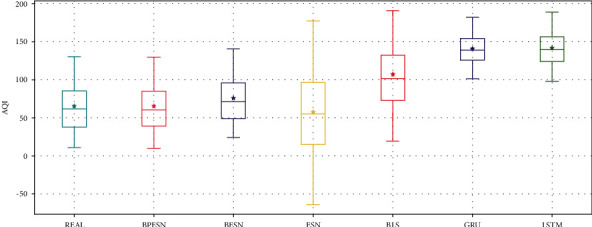
Boxplot of air quality datasets predicted by different network models.

The results of different methods are represented by boxplots, as shown in Figure 7. For the boxplots, the box body means the range of most of the data. It can be seen that the box body of ESN is the largest, indicating that the fluctuation of predicted data is the largest. BESN makes the information more concentrated, of which the mean and median are close to the actual data. BPESN performs the best among all the contrasts in view of the data distribution, median, and mean, indicating that the prediction ability of the BPESN model is the best.

To evaluate the performance of each model quantificationally, the performance evaluation indexes are calculated according to Section 4.1.1, as shown in Table 3. It can be seen that the BESN has a minor prediction error than the single ESN and BLS model. Referring to SMAPE, MAE, RMSE, and *R*^2^, the prediction error of BPESN is less than the other contrasts.

**Figure 8:**
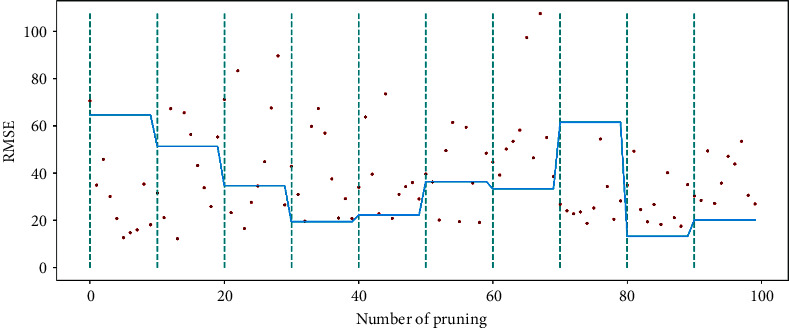
RMSE distribution of BESN before and after pruning on air quality dataset.

For the optimization capability of the pruning algorithm on the air quality dataset, Figure 8 shows the performance change of the BESN model before and after optimization by the pruning algorithm. The RMSE evaluation metric demonstrates the pruning effect. The lines between the two dashed lines represent the RMSE values obtained for the model without pruning optimization. Each scatter point between the two dashed lines represents the RMSE value of the model after one pruning optimization.

**Figure 9:**
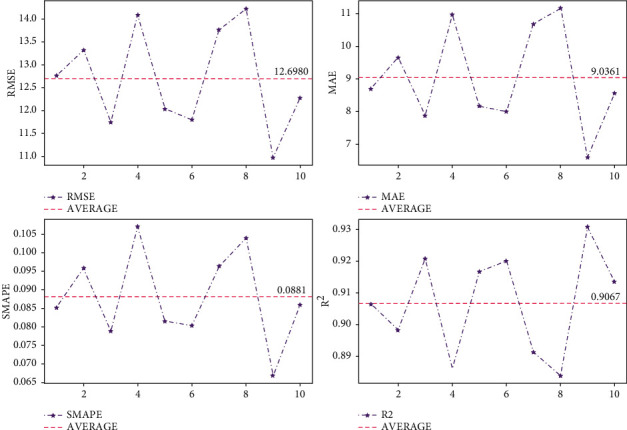
Cross-validation of BPESN on air quality dataset.

To validate the performance of BPESN on the air quality dataset, this experiment uses k-fold cross-validation (*k* = 10) for validation, and the evaluation metrics of each validation model are shown in Figure 9.

### 4.3. Prediction of Power Load Data

In the experiment of power load prediction, the parameters are set firstly to obtain the best performance of different models. The parameters of the networks are shown in Table 4.

**Figure 10:**
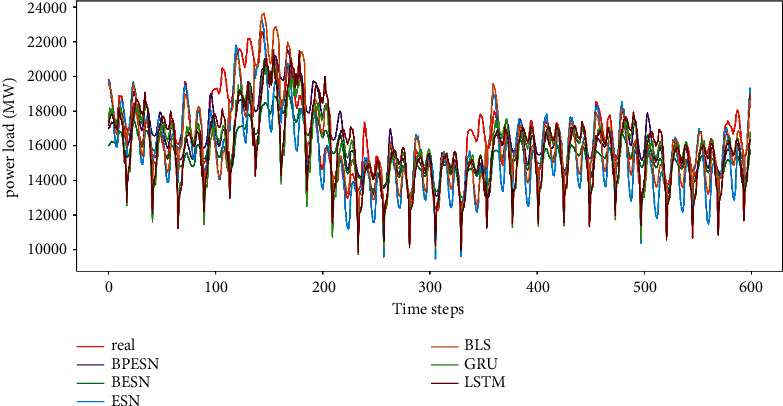
Prediction results of different network models in the US power load dataset.

The power load data is predicted with the proposed network and the contrast methods, as shown in Figure 10. The experimental results are denoted with lines in different colors, and it can be found that BPESN fits the actual data curve most closely. Meanwhile, it can also be seen that BESN has a significant improvement in the degree of fit compared to BLS and ESN. BPESN is the best in contrast after the pruning optimization.

**Figure 11:**
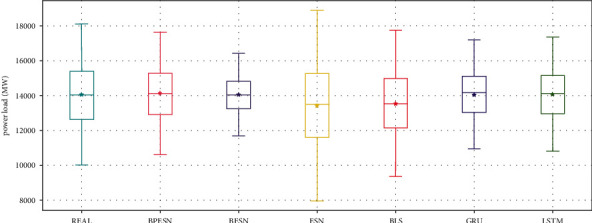
Boxplot distribution of the predicted results of different network models in the US power load dataset.

The distribution of the prediction results is shown in Figure 11. It can be seen that the ESN and BLS boxes are larger. The upper and lower caps cover the largest range in the ESN model, indicating that the forecast data fluctuates the most. The results of BPESN optimized through the pruning algorithm based on BESN are the closest to the actual data in terms of box size, mean, and median, indicating that the predictive ability of the BPESN model is the best among all comparison models.

The evaluation indicators are calculated in Table 5. The BESN network model has smaller prediction errors and more accurate capabilities than ESN, BLS, GRU, and LSTM. BPESN is a network based on BESN that has pruning optimization simultaneously. The SMAPE, MAE, and RMSE of BPESN descend compared with the BESN model. The fitting degree of the BPESN network is better in view of *R*^2^.

**Figure 12:**
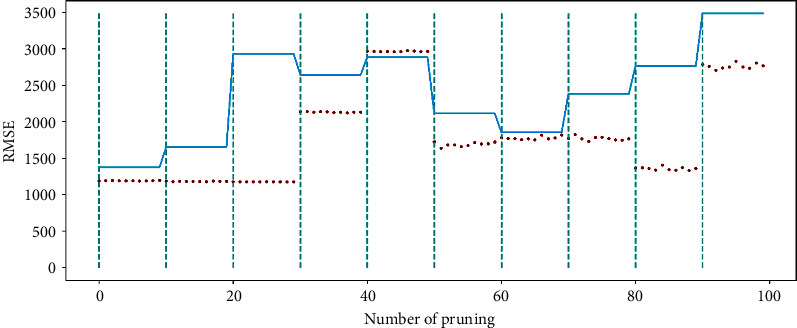
RMSE distribution of BESN before and after pruning on US power load dataset.

Figure 12 shows the optimization capability of the pruning algorithm on the US power load dataset, similar to Figure 8. The relationship between the dash locations and scatter points indicates that the BPESN network model has less prediction error than the BESN model. It can be seen that the pruning algorithm can still optimize the BESN model within a specific range.

**Figure 13:**
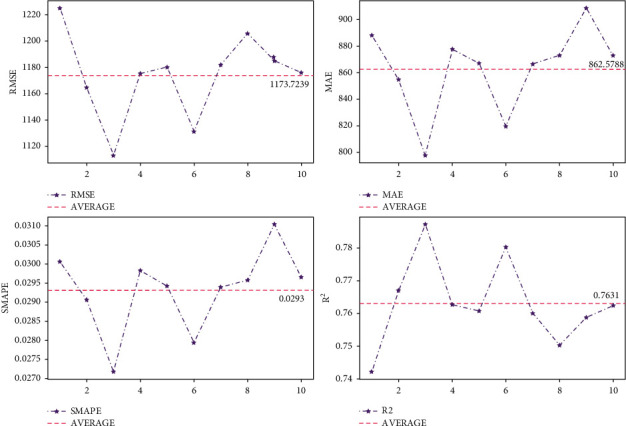
Cross-validation of BPESN on US power load dataset.

The cross-validation is also carried on BPESN for the electric load dataset. This experiment uses k-fold cross-validation (*k* = 10) for validation, and the evaluation metrics for each validation model are shown in Figure 13.

## 5. Discussion

### 5.1. Error Analysis

BPESN was proposed in this paper and verified in the experiments. It can be seen from Figures 7 and 11 that the box of ESN and BLS is more prominent, and the upper and lower cap of ESN have the most considerable coverage, indicating that the fluctuation of predicted data is the largest. Although the boxplot of BLS is smaller, the mean and median move back up. The box of the BESN model is smaller, and the median and mean move down appropriately. It can be seen from Tables 3 and 5 that the *R*^2^ of GRU and LSTM of BESN is closest to 1 relative to ESN, indicating that the network fitting ability of BESN is good. From the perspective of RMSE, BESN is 68.84%, 64.34%, 80.53%, and 80.46% lower than ESN, BLS, GRU, and LSTM, respectively. In terms of the American power load dataset, the RMSE of BESN decreases by 27.67%, 3.23%, and 8.22%, compared with ESN, GRU, and LSTM, respectively, but it increases by 5.42% compared with BLS. It can be seen from Figure 11 that BESN is not sensitive to fluctuations of power data, resulting in a smaller box and increased RMSE.

The pruning optimization algorithm acts on the reserved layer of ESN and realizes pruning through different contribution degrees of reservoir pool neurons, which can improve network performance to a certain extent and is verified by experiments. Figures 8 and 12, respectively, show the RMSE changes of the network before and after 100 pruning of BESN with the same configuration of BPESN. Suppose the RMSE after pruning is defined to be smaller than the RMSE before pruning. The effective pruning rate is 34% on the Fangshan air quality dataset and 90% on the American power load dataset. From the perspective of RMSE, the BPESN of Fangshan air quality dataset decreased by 19.35% compared with BESN, and the BPESN of the American power load dataset was reduced by 15.22%. From the perspective of *R*^2^, the network fitting ability of BPESN compared with BESN is improved by 5.36% and 13.87%, respectively. In terms of network model fitting degree and error, BPESN is superior to other models mentioned above.

To better evaluate the performance of network models and avoid the occurrence of chance phenomena, k-fold cross-validation (*k* = 10) is used to assess BPESN models. As shown in Figure 9, the RMSE and *R*^2^ fluctuate between the intervals [10.97, 14.22] and [0.88, 0.93] with mean values of 12.698 and 0.9067, respectively. As shown in Figure 13, the RMSE and *R*^2^ fluctuate between the intervals [1112.96, 1225.04] and [0.74, 0.78] with mean values of 1173.7239 and 0.7631, respectively. In the cross-validation, each evaluation index fluctuates less around the mean value. It indicates that BPESN has better prediction performance and stability on the Fangshan air quality and US power load datasets.

### 5.2. Complexity Analysis

The complex network computation and structure can reduce the prediction error and improve the prediction performance, which usually increases time and space consumption. This subsection will analyze the complexity of the models based on ESN, including the time complexity and space complexity. Assuming *K* > *N*, the running time of each ESN is dominated by the reservoir pool computation Ο(*KN*^2^) and the pseudo-inverse computation Ο(*N*^3^) [37]. The space occupation is concentrated in the reservoir pool computation process, and the space complexity is Ο(*N*^2^). Then, the time complexity of the ESN is shown in as follows:

(11) $$\begin{array}{c} Ο\left(KN^{2}+N^{3}\right)\approx Ο\left(KN^{2}\right). \end{array}$$

The mapping layer in BESN is a linear matrix operation with time complexity Ο(*n*), and there are *m* ESN units in the reinforcement layer with time complexity Ο(*m*(*KN*^2^+*N*^3^)). The space occupation is concentrated in the computation of the reservoir pool in the reinforcement layer. Then, the time complexity of BESN is shown as follows:

(12) $$\begin{array}{c} Ο\left(n+m\left(KN^{2}+N^{3}\right)\right)\approx Ο\left(m\left(KN^{2}+N^{3}\right)\right)\approx Ο\left(mKN^{2}\right). \end{array}$$

BPESN is implemented based on BESN by the pruning optimization algorithm. The time complexity of the optimization algorithm is Ο(2*PN*^2^log  *N*), and *P* is the number of pruning. The space complexity is Ο(*mN*^2^) and the time complexity of BPESN is shown in equation (13). The time and space complexity of different ESN-based networks are summarized in Table 6.

(13) $$\begin{array}{c} Ο\left(n+m\left(KN^{2}+N^{3}+2PN^{2}\log\;N\right)\right)\\ \approx Ο\left(m\left(KN^{2}+N^{3}+2PN^{2}\log\;N\right)\right)\\ \approx Ο\left(m\left(KN^{2}+2PN^{2}\log\;N\right)\right). \end{array}$$

While the accuracy of the network model is improved, it can be seen from Table 6 that the time and space complexity of BPESN are larger than those of a single ESN and BESN. This is due to the special structure of the broad learning system and the pruning optimization algorithm. BPESN needs more time and space to learn a network structure to improve network performance.

## 6. Conclusion

A new network structure is studied in this paper. The proposed BPESN integrates the ESN in the framework of BLS with the pruning algorithm. In the optimization, the correlation coefficient matrix of the neuron in the reservoir pool is calculated, based on which the network model can remove the redundant information. The network's fitting ability is improved, and a better prediction effect is achieved. In the experiment, the datasets are tested with the nonstationary evaluation method. It is proved that the proposed network applies to the nonstationary time series data. Based on the experiment verification in this paper, the proposed network should be validated in the theoretical analysis and practical applications in future work [43–46].

## Figures and Tables

**Algorithm 1:**
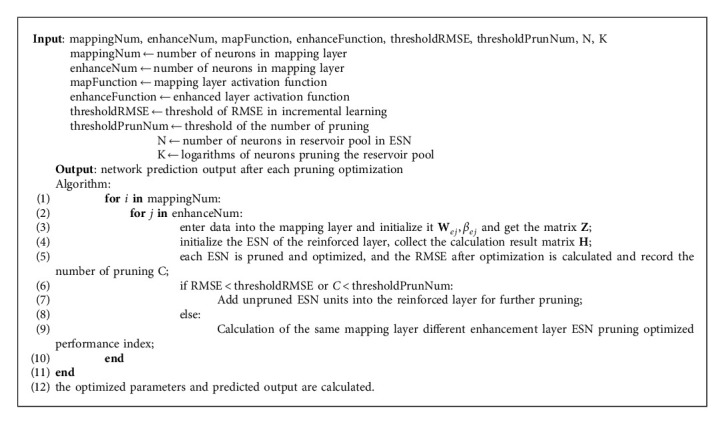
BPESN with pruning optimization.

**Table 1:** Nonstationary degree tests based on ADF.

Dataset	*p*	*TS*	*CV*_1_	*CV*_5_	*CV*_10_
Air quality dataset of Fangshan District in Beijing	0.1608	−2.3357	−3.4325	−2.8623	−2.5672
US power load	0.3550	−2.4469	−3.9602	−3.4109	−3.1272

**Table 2:** Network model parameters of Fangshan District air quality dataset in Beijing.

Hyperparameter	BPESN	BESN	ESN	BLS
Number of neurons in mapping layer	1–15	1–15	N/A	1–15
Number of neurons in enhancement layer	1–20	1–20	N/A	1–20
Reservoir size	400–800	400–800	400–800	N/A
Spectral radius rate	0.95	0.95	0.95	N/A
Leaking rate	0.1	0.1	0.1	N/A
Sparseness	0.05	0.05	0.05	N/A
Pruning the number of times	1–10	N/A	N/A	N/A

**Table 3:** Evaluation indexes of each model of Fangshan District air quality dataset in Beijing.

Model	Training time	SMAPE	MAE	RMSE	*R* ^2^
ESN	3.3921	0.35804	33.08395	48.83188	−0.36982
BLS	0.0471	0.26211	39.80818	42.67138	−0.04600
GRU	121.6488	0.40606	74.78422	78.14060	−2.50762
LSTM	113.3026	0.40862	75.36049	77.88657	−2.48485
BESN	46.6092	0.12338	12.87887	15.21721	0.86697
BPESN	82.3575	0.08593	8.56364	12.27334	0.91346

**Table 4:** Network model parameters of the US power load dataset.

Hyperparameter	BPESN	BESN	ESN	BLS
Number of neurons in mapping layer	1–15	1–15	N/A	1–15
Number of neurons in enhancement layer	1–20	1–20	N/A	1–20
Reservoir size	500–1000	500–1000	500–1000	N/A
Spectral radius rate	0.95	0.95	0.95	N/A
Leaking rate	0.1	0.1	0.1	N/A
Sparseness	0.05	0.05	0.05	N/A
Pruning the number of times	1–10	N/A	N/A	N/A

**Table 5:** Evaluation indexes of each model in the US power load dataset.

Model	Training time	SMAPE	MAE	RMSE	*R* ^2^
ESN	3.8098	0.0529	1453.4936	1915.5536	0.3696
BLS	0.1380	0.0332	909.3070	1311.3357	0.7045
GRU	169.8198	0.0390	1127.0565	1432.7877	0.6473
LSTM	152.8951	0.0410	1190.1022	1510.7332	0.6079
BESN	77.2083	0.0358	1055.7738	1386.5070	0.6697
BPESN	109.9256	0.0297	875.2953	1175.4368	0.7626

**Table 6:** Time complexity and space complexity of different models.

Model	Time complexity	Space complexity
ESN	Ο(*KN*^2^)	Ο(*N*^2^)
BESN	Ο(*mKN*^2^)	Ο(*mN*^2^)
BPESN	Ο(*m*(*KN*^2^+2*PN*^2^log *N*))	Ο(*mN*^2^)

## Data Availability

The data used to support the findings of this study are available from the corresponding author upon request.
